# Metal Complexes with Schiff Bases: Data Collection and Recent Studies on Biological Activities

**DOI:** 10.3390/ijms232314840

**Published:** 2022-11-27

**Authors:** Maria Stefania Sinicropi, Jessica Ceramella, Domenico Iacopetta, Alessia Catalano, Annaluisa Mariconda, Camillo Rosano, Carmela Saturnino, Hussein El-Kashef, Pasquale Longo

**Affiliations:** 1 Department of Pharmacy, Health and Nutritional Sciences, University of Calabria, 87036 Arcavacata di Rende, Italy; 2 Department of Pharmacy-Drug Sciences, University of Bari “Aldo Moro”, 70126 Bari, Italy; 3 Department of Science, University of Basilicata, 85100 Potenza, Italy; 4 Proteomics and Mass Spectrometry Unit, IRCCS Policlinico San Martino, Largo Rosanna Benzi, 10, 16132 Genoa, Italy; 5 Department of Chemistry, Faculty of Science, Assiut University, Assiut 71516, Egypt; 6 Department of Chemistry and Biology, University of Salerno, Via Giovanni Paolo II, 132, 84084 Fisciano, Italy

**Keywords:** Schiff bases, antibacterial, imine, antimicrobial, metal complexes, antiproliferative, antimalarial, antileishmanial, Alzheimer disease, diabetes

## Abstract

Metal complexes play a crucial role in pharmaceutical sciences owing to their wide and significant activities. Schiff bases (SBs) are multifaceted pharmacophores capable of forming chelating complexes with various metals in different oxidation states. Complexes with SBs are extensively studied for their numerous advantages, including low cost and simple synthetic strategies. They have been reported to possess a variety of biological activities, including antimicrobial, anticancer, antioxidant, antimalarial, analgesic, antiviral, antipyretic, and antidiabetic ones. This review summarizes the most recent studies on the antimicrobial and antiproliferative activities of SBs-metal complexes. Moreover, recent studies regarding mononuclear and binuclear complexes with SBs are described, including antioxidant, antidiabetic, antimalarial, antileishmanial, anti-Alzheimer, and catecholase activities.

## 1. Introduction

**Figure 1:**
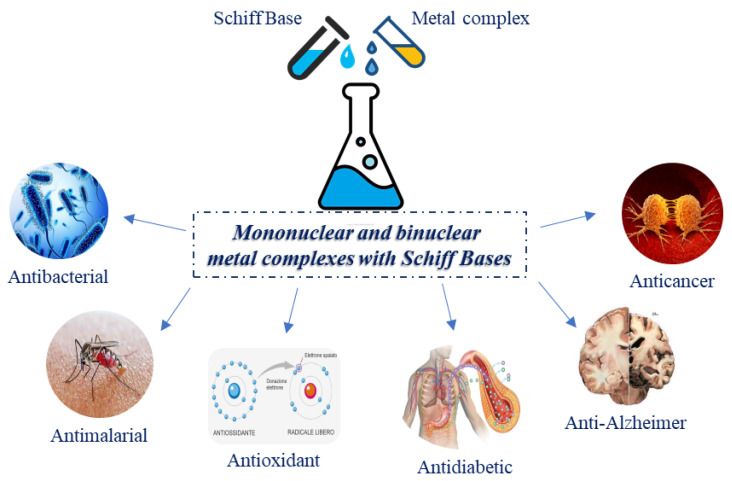
Multiple activities of mononuclear and binuclear metal complexes with Schiff Bases.

Coordination chemistry is an attractive discipline for chemists and researchers who have reached pharmaco-therapeutic success in the field of remediation of severe diseases and improvements of several drugs. SBs are versatile organic compounds deriving from the condensation of primary amines with carbonyl compounds; they contain an imine group (azomethine, >C=N–). In addition to their usefulness in catalysis [1], they showed diverse biological activities [2,3] including antimicrobial [4], antiproliferative [5], antimalarial [6,7], analgesic, anxiolytic [8], antidepressant [9], anti-inflammatory [10,11], antiviral [12], antipyretic, antibacterial [13] and antifungal [14]. Moreover, several studies on SBs-metal complexes showed their use as anti-Alzheimer agents [15,16,17], and their potential in the management of diabetes mellitus [18]. Some SBs derivatives have demonstrated α-glucosidase inhibition and antiglycation activity [19,20], whereas other derivatives were synthesized and used as fluorescent sensors for the diabetic biomarker beta-hydroxybutyrate (β-HB) [21]. Metal complexes of different metals with SBs have demonstrated a myriad of activities [22,23], and catalytic applications [24] in which metals are represented by Mo [25], Cu [26,27,28], Zr [29,30,31], Pd [32], Cu [33], Ni [34], Ru [35], Mn [36], Zn [37,38] and W [39]. Several studies have been carried out on the transmetalation of Zn-SB complexes with other metal ions [40] for various purposes, such as the detection of Cu^2+^ ions in aqueous solution [41] and the inhibition of acid-induced steel corrosion [42]. Recent studies addressed luminescence and fluorescence taking advantage of lanthanide (Ln^3*+*^) complexes with SBs [43]. It is noteworthy that not only mononuclear but also binuclear metal complexes with SBs have recently attracted attention in diverse fields of research (Figure 1). Chi et al. [44] have studied a Zn-Yb binuclear SBs complex, which enhanced near-infrared (NIR) luminescence. A ytterbium SBs complex was suggested for NIR-emitting organic light emitting diode (OLED), a technology recently emerged for its use in a vast range of applications such as medical diagnostics (oximetry, drug delivery, tumor therapy, atherosclerosis treatment) [45]. Fluorescence properties were found for mononuclear Dy(III)/heteropolynuclear Zn(II)–Dy (III) SBs [46]. Nano SBs are considered the most multitasking ligands as they are able to form complexes with metals [47]. SBs-metal complexes are frequently used as environmentally friendly catalysts [48]: Ni-SBs complexes have been used for green catalysis [49], whereas a copper (II) SBs complex was fixed on the surface of iron oxide nanoparticles, which was used for oxidation of olefins with H_2_O_2_ as an eco-friendly oxidant [50]. Despite the importance of all these activities, we would like to focus on the biological aspects of SBs-metal complexes. Indeed, various biological activities have been widely described for SBs-metal complexes, including antimicrobial [51,52], antioxidant, anticancer [53], and some DNA interaction studies [54,55,56]. The antiproliferative activity of SBs-metal complexes has been recently reviewed [57,58]. In this review, the most recent studies on these complexes are summarized, mainly focusing on the antimicrobial activity, which is one of the main challenges, currently, to overcome antimicrobial resistance [59]. For binuclear complexes, most of the studies are also in relation to antiproliferative, antioxidant, and catecholase activities.

## 2. Mononuclear SBs Complexes

### 2.1. Mononuclear SBs Complexes with Antibacterial and Antiproliferative Activities

The antimicrobial and antiproliferative activities of some mononuclear SBs complexes are reported in Table 1. Generally, the data refer to the lowest concentration of the assayed antimicrobial agent (minimal inhibitory concentration, MIC) that, under defined test conditions, inhibits the visible growth of the bacterium being investigated. Microbes were generally referred to in either the American Type Culture Collection (ATCC), Persian Type Culture Collection (PTCC), or Agricultural Culture Collection of China (ACCC), unless otherwise detailed. Several authors determined the antimicrobial activity by measuring the diameter (as mm) of the zone showing the complete inhibition (inhibition zone diameter, IZD). In one or more cases, the bacterial suspensions were prepared via the direct colony method. At the end of the assay, the number of survived bacterial colonies (measured as CFUs) was quantified and the rates of colony-forming units (R) were calculated as a percentage with respect to the control, which contained no antibacterial agent. Cytotoxicity studies were reported giving IC_50_ values (the concentration that kills or inhibits the cell viability by 50%).

Rajakkani et al., (2021) [60] reported the synthesis and biological evaluation of stable mononuclear metal (II) complexes (specifically, Co, Ni, and Zn) with an unsymmetrical 13-membered Knoevenagel macrocyclic SB ligand. Antimicrobial activity was determined by using the well dilution method against bacteria (two Gram-positive, *Staphylococcus aureus*, *Bacillus subtilis*, and three Gram-negative, *Escherichia coli*, *Klebsiella pneumoniae*, *Salmonella typhi*) compared with the standard kanamycin (MIC = 1.4, 1.2, 1.8, 2.2 and 2.0 µM, respectively) and fungi (*Aspergillus niger*, *Fusarium solani*, *Aspergillus flavus*, *Rhizoctonia bataticola*, *Candida albicans*) compared with the standard fluconazole (MIC = 1.2, 1.5, 1.2, 1.8 and 1.8 µM, respectively). The interesting results of the complex [CuL]Cl_2_ (**1**), higher than those obtained with the ligand, were justified by the chelation theory, in which the chelation decreases the polarity of the metal ion partially sharing its positive charge with donor groups of ligand and the potential π electron delocalization may be found around each ring. Moreover, antiproliferative assays were carried out against human breast (MCF-7), cervical (HeLa), and epithelioma (Hep-2) cancer cell lines, as well as against the normal human dermal fibroblast (NHDF) cell line, by the MTT assay method. Indeed, higher anticancer activity of Cu(II) complex was interesting as compared to cisplatin (IC_50_ = 15 ± 1.2, 18 ± 1.2, 14 ± 1.0 µM, and 82 ± 1.0 µM, respectively).

Abu-Dief et al., (2021) [61] studied a series of metal complexes derived from Zn(II), Pd(II), Cr(III), and VO(II) with an SB derivative (HNP) [HNP = 1-(pyrimidin-2-yliminomethyl)-naphthalen-2-ol] for their antimicrobial activities against two Gram-negative (*Serratia marcescens* and *E. coli*) and one Gram-positive (*Micrococcus luteus*) bacteria, and fungal species (*Fusarium oxysporum*, *Aspergillus flavus*, and *Geotrichum candidum*). HNPPd (**2**) showed a significant inhibition that was similar to the standards (ofloxacin IZD = 31 ± 0.21 mm, 26 ± 0.17 mm, and 42 ± 0.30 mm, respectively, against bacteria) and fluconazole (IZD = 21 ± 0.17 mm against *A. flavus*). The antiproliferative activity was studied against three human cancer cell lines, human colon cancer (HCT-116), MCF-7, and human hepatocellular carcinoma (HepG-2), comparing to vinblastine (IC_50_ = 4.12 ± 0.11, 3.3 ± 0.10, and 7.5 ± 0.08, respectively). Complex **2** showed good antiproliferative activity by decreasing the growth of cancer cells. 

Kargar et al., (2021) [62] studied several Ni(II), Cu(II), and Zn(II) complexes with ONNO donor salen-type SB ligands, derived from different 3,5-dihalosalicylaldehyde with polymethylenediamines of variable chain length and the most interesting antimicrobial compounds were ZnL2 (**3**), NiL3 (**4**) and CuL3 (**5**). The antibacterial activity was evaluated against two Gram-positive (*S. aureus* PTCC 1431 and *Bacillus cereus* PTCC 1015) and two Gram-negative (*E. coli* PTCC 1394 and *Pseudomonas aeruginosa* PTCC 1074) bacterial strains by using the disc diffusion method. The complexes showed activity against all the strains tested compared with the standards, erythromycin (IZD = 16, 17, 25, 24 mm, respectively) and ampicillin (IZD = 11, 14, 27, 26 mm, respectively). Complex **4**, which was the most active, was characterized by single crystal X-ray diffraction, showing that it adapted slightly distorted octahedral geometries around Ni(II).

Kargar et al., (2022) [63] described the synthesis of a nickel(II) complex (NiLUns, **6**) prepared by reacting an unsymmetrical tetradentate salophen-based SB with Ni(CH_3_COO)_2_·4H_2_O. By studying the crystal structure of the nickel complex with X-ray diffraction it was evidenced that the ligand chelates the Ni(II) ion by using its ONNO set of donor sites forming a square planar geometry. The antibacterial activity was evaluated against *S. aureus* PTCC 1431, *B. cereus* PTCC 1015, *E. coli* PTCC 1394, and *P. aeruginosa* PTCC 1074 bacterial strains by using the disc diffusion method. Complex **6** showed activity against all the strains tested compared with the standards, erythromycin (IZD = 16, 17, 25, 24 mm, respectively) and ampicillin (IZD = 11, 14, 27, 26 mm, respectively). The authors speculated that the increased activity of the metal complex was due to its ability to interfere with cell respiration and protein synthesis, inducing organism death.

Al-Shboul et al., (2022) [64] reported an interesting study on metal complexes (Fe, Cu, and Zn) of substituted salicylideneamino-4,4′-dimethyl-1,1′-biphenyl SBs, which show differences of activities depending on the coordinating metal. Complexes with zinc, Z2Zn (**7**), Z3Zn (**8**), and Z4Zn (**9**), showed antimicrobial activity against Gram-positive bacteria (*M. luteus* ATCC 934 and *S. aureus* 29213), compared with amoxicillin (IZD = 25 mm and 35 mm, respectively) whereas complexes with copper, Z1Cu (**10**) and Z3Cu (**11**), showed activity against the Gram-negative *E. coli* ATCC25922 (IZD = 10 mm for amoxicillin). All complexes were also tested for their antiproliferative activities against parental breast cancer (MCF7) [65], non-small-cell lung cancer (A549), and human dermal fibroblast (HDF) cell lines, compared to doxorubicin (0.03 µg/mL, 0.15 µg/mL and 0.37 µg/mL, respectively) via the MTT assay. The highest antiproliferative activity was found for the copper complex Z2Cu (**12**). 

Maia et al., (2022) [66] recently described the synthesis of a nickel(II) chloride SB complex [(Ni(L2), **13**] and its antimicrobial activity evaluation against multi-resistant *S. aureus* 10 (from catheter tip), *E. coli* 06 (from urine culture) and *P. aeruginosa* 15 (from rectal swab) strains and leishmania. This complex showed inhibition of the bacterial growth for the strains of *P. aeruginosa*, at the concentration of 256 μg/mL, while it was not active against *E. coli* and *S. aureus* (MIC ≥ 1024 μg/mL). The modulation of the antibiotic activity against multi-resistant strains was also studied, indeed complex **13** increased the activity of gentamicin against *S. aureus*, decreasing the MIC from 50.8 to 16 μg/mL that, proportionally, corresponds to a 68.5% reduction in the amount of gentamicin needed to produce the same effect on the strain under study. For *E. coli,* the decrease was about 20.6% (MIC from 32 to 25.4 μg/mL). Moreover, complex **13** increased the activity of amikacin against *E. coli* with a 37% reduction (MIC from 203.2 to 128 μg/mL). Thus, the same complex in combination with gentamicin or amikacin determined a decrease in the antimicrobial activity in both cases. The compound also showed interesting results against *Leishmania amazonensis* promastigote via the Promastigotes assay, by measuring the culture density after 24, 48, and 72 h of incubation and counting the viable parasites in a Neubauer chamber, in order to determine the IC_50_. 

Tyula et al., (2023) [67] reported studies on SB complexes with metals (Zn^2+^, Cd^2+^, Hg^2+^, and Cd^2+^) and their antibacterial activities. In-depth studies for determining the structure were carried out on the Cd(II) complex, [Cd(H_2_L)]·2H_2_O (**14**), (H_4_L = *N,N,N’,N’*-tetrakis (5-bromo salicylidene) *N,N,N’,N’*-tetrakis(2-aminoethyl)ethylenediamine) by single crystal X-ray crystallography and Hirshfeld surface analysis with the corresponding 2D fingerprint plots. Antibacterial activities of the compounds were tested by the disc diffusion method against three Gram-positive bacteria (*S. aureus* ATCC 25923, *E. faecalis* ATCC 23212, and *Staphylococcus epidermidis* ATCC 34384), and three Gram-negative bacteria (*E. coli* ATCC 25922, *P. aeruginosa* ATCC 27853, and *K. pneumoniae* ATCC 70063). All the compounds demonstrated moderate antibacterial activities and the most active was the Hg(II) complex (**15**), which was more active than the standard sulfisoxazole (300 µg/mL, IZD = 9 mm (*P. aeruginosa* ATCC 27853, 200 μg/disk); IZD = 17 mm (*E. faecalis* ATCC 23212, 200 μg/disk); IZD = 15 mm (*S. epidermidis* ATCC 34384, 200 μg/disk). When determining the percentage of inhibition, all the complexes showed excellent activity against *P. aeruginosa* (120–200% inhibition). The Hg complex also exhibited a good activity against *E. coli* (90% inhibition); and an excellent activity against *S. epidermidis* and *E. faecalis* (120–140% inhibition). 

Saritha et al., (2021) [68] studied a SB ligand with structure > C=N–N– derived from curcumin and semicarbazide and its transition metal [Mn(II), Co(II), Ni(II), Cu(II), and Zn(II)] complexes for their antimicrobial and anti-inflammatory activity. The antimicrobial activity was assessed against bacteria (*B. subtilis*, *S. aureus*, *E. coli*, *K. pneumoniae,* and *P. aeruginosa*) and fungi (*C. albicans* and *A. niger*) by the agar well diffusion method. The Zn complex Zn(HMHC)_2_]H_2_O (**16**) showed high activity against the bacteria *K. pneumoniae* and *B. subtilis* with respect to the standard amikacin (IZD = 24 and 22 mm, respectively) and against fungi compared to nystatin (*C. albicans* and *A. niger*, IZD = 15 and 17 mm, respectively). The enhanced activity of the complexes was explained on the basis of the Overtone’s concept [69] and Tweedy’s Chelation theory [70]. Moreover, the anti-inflammatory activity of complexes was studied by using the inhibition of albumin denaturation technique, according to a partially modified literature procedure [71]. Complex **16** showed activity in inhibiting the heat-induced albumin denaturation at different concentrations, showing a maximum inhibition of 92.2% at a concentration of 100 μg/mL, compared to diclofenac sodium (100% inhibition).

Gowdhami et al. (2022) [72] recently described the antiproliferative activity of cobalt(III) SB complexes (**17** = Co(1-phenylbutane-1,3-dione)(heptylamine)_2_](-ClO_4_) and **18** = [Co(1-phenylbutane-1,3-dione)(dodecyl amine)_2_](-ClO_4_)] against human breast MCF-7 and lung A549 cancer cell using the MTT cell viability assay. The two complexes showed high antiproliferative activity compared to the standard cisplatin (IC_50_ = 11.6 ± 0.1 µM and 14.4 ± 0.1 µM, respectively).

An interesting article has been recently reported by Hassona et al. [73] regarding the anticancer activity of a Pd complex with an SB the palladium(II) 2-hydroxyimino-3-(2-hydrazonopyridyl)-butane [PdLCl]·H_2_O (**19**). This study investigated the antitumor and apoptotic activities of the complex against Ehrlich carcinoma in vitro and in vivo. Ehrlich ascites carcinoma (EAC) cells were used, and caspase 8 activity was estimated using the Caspase 8 FAM-FLICA kit. In vitro, the complex reduced the EAC cells’ viability, enhanced the caspase 8 activity, arrested the cell cycle at G0/G1, and reduced the G2(M) population, indicating that the antiproliferative and antitumor activities were likely due to the induction of apoptosis. In vivo studies were carried out on adult female Swiss albino mice, divided into different groups: normal, EAC, EAC+cisplatin, EAC+complex, and normal+complex. Treatment with the complex in a dose-dependent mode significantly diminished the tumor volume and weight, prolonged the median survival time (MST) and the percent increase in life span (ILS%), enhanced the mice body weight gain, and improved the blood indexes. Treatment of EAC-bearing mice with the complex highest dose showed more desirable outcomes than the treatment with cisplatin, moreover, the normal+complex group showed no pathological changes, indicating safety.

**Table 1:** SBs mononuclear metal complexes with antimicrobial and antiproliferative activities.

Structure	Compound	Antimicrobial Activity (MIC or IZD)	IC50	Ref
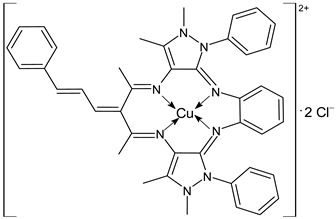	[CuL]Cl_2_ (**1**)	MIC = 5.6 µM (*S. aureus*) MIC = 5.8 µM (*B. subtilis*) MIC = 5.4 µM (*E. coli*) MIC = 6.2 µM (*K. pneumoniae*) MIC = 5.8 µM (*S. typhi*) MIC = 5.8 µM (*A. niger*) MIC = 5.6 µM (*F. solani*) MIC = 5.2 µM (*A. flavus*) MIC = 6.4 µM (*R. bataticola*) MIC = 6.2 µM (*C. albicans*)	IC_50_ = 14 ± 0.8 µM (HeLa) IC_50_ = 16 ± 1.0 µM (MCF-7) IC_50_ = 16 ± 1.0 µM (Hep-2) IC_50_ = 86 ± 1.0 µM (NHDF)	Rajakkani et al., 2021 [60]
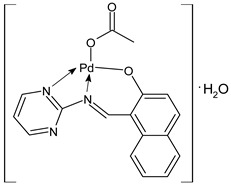	HNPPd (**2**)	MIC = 3.25 µM; IZD = 28 ± 0.13 mm (*S. marcescens*) MIC = 3.75 µM; IZD = 22.5 ± 0.09 mm (*E. coli*) MIC = 3.00 µM; IZD = 38.5 ± 0.12 mm (*M. luteus*) IZD = 19 ± 0.09 mm (*A. flavus*) IZD = 35.0 ± 0.08 mm (*G. candidum*) IZD = 22.5 ± 0.14 mm (*F. oxysporum*)	IC_50_ = 6.75 ± 0.08 (HCT-116) IC_50_ = 17.85 ± 0.10 (MCF-7) IC_50_ = 13.10 ± 0.15 (HepG-2)	Abu-Dief et al., 2021 [61]
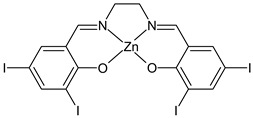	ZnL2 (**3**)	IZD = 13 mm (*E. coli* PTCC1394) IZD = 15 mm (*P. aeruginosa* PTCC1074) IZD = 29 mm (*S. aureus* PTCC1431) IZD = 26 mm (*B. cereus* PTCC1015)	-	Kargar et al., 2021 [62]
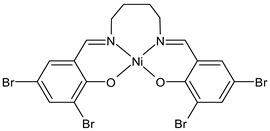	NiL3 (**4**)	IZD = 13 mm (*E. coli* PTCC1394) IZD = 15 mm (*P. aeruginosa* PTCC1074) IZD = 29 mm (*S. aureus* PTCC1431) IZD = 26 mm (*B. cereus* PTCC1015)	-	Kargar et al., 2021 [62]
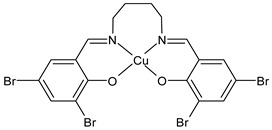	CuL3 (**5**)	IZD = 16 mm (*E. coli* PTCC1394) IZD = 14 mm (*P. aeruginosa* PTCC1074) IZD = 23 mm (*S. aureus* PTCC1431) IZD = 26 mm (*B. cereus* PTCC1015)	-	Kargar et al., 2021 [62]
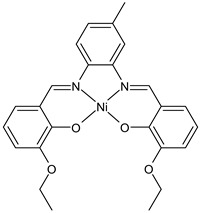	NilUns (**6**)	IZD = 15 mm (*E. coli* PTCC1394) IZD = 14 mm (*P. aeruginosa* PTCC1074) IZD = 25 mm (*S. aureus* PTCC1431) IZD = 21 mm (*B. cereus* PTCC1015)	-	Kargar et al., 2021 [63]
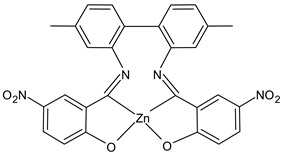	Z2Zn (**7**)	IZD = 15 mm (*M. luteus* ATCC 934) IZD = 21 mm (*S. aureus* ATCC 29213)	IC_50_ = 25.2 µg/mL (MCF-7) IC_50_ = 81.2 µg/mL (A549) IC_50_ = 68.7 µg/mL (HDF)	Al-Shboul et al., 2022 [64]
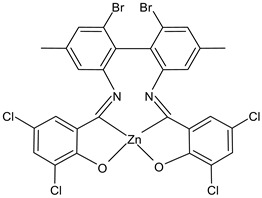	Z3Zn (**8**)	IZD = 25 mm (*M. luteus* ATCC 934) IZD = 18 mm (*S. aureus* ATCC 29213)	IC_50_ = 17.7 µg/mL (MCF-7) IC_50_ = 199.4 µg/mL (A549) IC_50_ = 27.6 µg/mL (HDF)	Al-Shboul et al., 2022 [64]
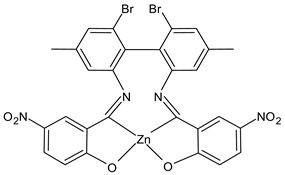	Z4Zn (**9**)	IZD = 15 mm (*M. luteus* ATCC 934) IZD = 25 mm (*S. aureus* ATCC 29213)	IC_50_ = 14.1 µg/mL (MCF-7) IC_50_ = 25.2 µg/mL (A549) IC_50_ = 28.3 µg/mL (HDF)	Al-Shboul et al., 2022 [64]
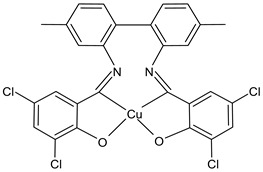	Z1Cu (**10**)	IZD = 10 mm (*E. coli* ATCC25922)	IC_50_ = 10.4 µg/mL (MCF-7) IC_50_ = 43.7 µg/mL (A549) IC_50_ = 8.5 µg/mL (HDF)	Al-Shboul et al., 2022 [64]
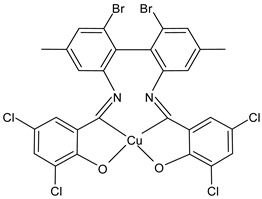	Z3Cu (**11**)	IZD = 20 mm (*E. coli* ATCC25922)	IC_50_ = 172.0 µg/mL (MCF-7) IC_50_ = 130.0 µg/mL (A549) IC_50_ = 92.0 µg/mL (HDF)	Al-Shboul et al., 2022 [64]
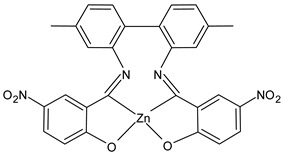	Z2Cu (**12**)	-	IC_50_ = 1.9 µg/mL (MCF-7) IC_50_ = 4.0 µg/mL (A549) IC_50_ = 1.5 µg/mL (HDF)	Al-Shboul et al., 2022 [64]
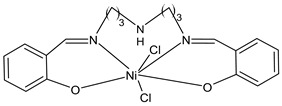	Ni(L2) (**13**)	MIC = 256 µg/mL (*P. aeruginosa* 15) MIC = 50.8→16 μg/mL (gentamicin *S. aureus*) MIC = 32→25.4 μg/mL (gentamicin *E. coli*) MIC = 203.2→128 μg/mL (amikacin *E. coli*)	IC_50_ = 1.076 ± 0.04039 μg/mL after 72 h (*L. amazonensis*)	Maia et al., 2022 [66]
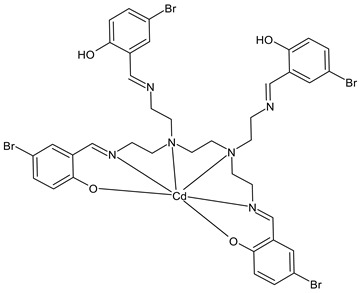	Cd(H2L) (**14**)	IZD = 11 mm (*P. aeruginosa* ATCC 27853, 200 μg/disk) IZD = 11 mm (*E. faecalis* ATCC 23212, 200 μg/disk) IZD = 11 mm (*S. epidermidis* ATCC 34384, 200 μg/disk)	-	Tyula et al., 2023 [67]
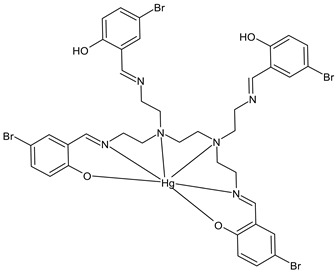	Hg(H2L) (**15**)	IZD = 13 mm (*P. aeruginosa* ATCC 27853, 200 μg/disk) IZD = 20 mm (*E. faecalis* ATCC 23212, 200 μg/disk) IZD = 17 mm (*S. epidermidis* ATCC 34384, 200 μg/disk)	-	Tyula et al., 2023 [67]
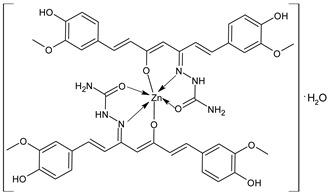	[Zn(HMHC)_2_]H_2_O (**16**)	IZD = 23 mm (*B. subtilis*) IZD = 23 mm (*K. pneumoniae*) IZD = 15 mm (*C. albicans*) IZD = 17 mm (*A. niger*) Percentage inhibition = 92.2% (Protein denaturation)	-	Saritha et al., 2021 [68]
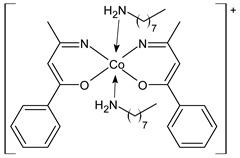	**17**	-	IC_50_ = 1.6 ± 0.1 µM (MCF-7) IC_50_ = 9.6 ± 0.1 µM (A549)	Gowdhami et al., 2022 [72]
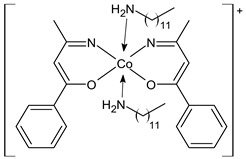	**18**	-	IC_50_ = 2.9 ± 0.2 µM (MCF-7) IC_50_ = 9.1 ± 0.1 µM (A549)	Gowdhami et al., 2022 [72]
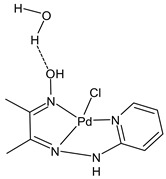	[PdLCl]·H_2_O (**19**)	-	IC_50_ = 3.4 × 10^–6^ μg/mL (EAC)	Hassona et al., 2022 [73]

### 2.2. Mononuclear SBs Complexes with Antimalarial, Antioxidant, Antidiabetic, and Anti-Alzheimer Activities

Shaikh et al., (2022) [74] recently studied two SBs zinc complexes with the structure -C=N-N- for their antimalarial activities against *P. falciparum* using the MTT assay. The complex Zn(BZ-PCBPMP)_2_ (**20**) with (*Z*)-*N*’-((4-chlorophenyl)-(1-phenyl)-3-methyl-5-oxo-1,5-dihydro-4*H*-pyrazol-4-ylidene) methyl) benzohydrazide) was the most active compared with the standards, quinine (MIC = 0.8 μmol/L) and chloroquine showing (MIC = 0.09 μmol/L).

Ragole et al., (2022) [75] studied Mn(II), Fe(III), Co(II), Ni(II), and Cu(II) complexes with the SB (*E*)-2-((4-chloro-3-nitrophenylimino)(phenyl)methyl)-5-methoxyphenol for their antibacterial, antimalarial, antioxidant, antidiabetic, and anticancer activities. The most interesting antimalarial activity was found for Fe(III) complex (**21**), with a 65.54% inhibition value that is close to the value of the standard chloroquine diphosphate 68.90%. The results were surprising for the antidiabetic activity, which was tested by the α-amylase inhibition assay. In this assay, all the complexes exhibited values of inhibition in the range of 53.03–55.55%, whereas the SB ligand was more active (59.09%), and the standard acarbose showed 80.80% inhibition. Moreover, antioxidant studies were carried out via DPPH assay, indicating that the Fe(III) complex **21** scavenged the DPPH radicals close to acarbose (97.79%).

Şenocak et al., (2021) [76] studied three SBs Zn(II) complexes with amino acids for their potential activities in diabetes and Alzheimer disease. The activity for Alzheimer disease was evaluated in comparison with the standard tacrine, by measuring the activity on the AChE (IC_50_ = 25.72 µM and *K*_i_ = 23.11 ± 4.05 µM) and BChE (IC_50_ = 18.06 µM and *K*_i_ = 14.10 ± 5.62 µM). The three complexes effectively inhibited the AChE, being more active than the standard; the major activity was found for complexes **22** and **23**. Moreover, the inhibitory effect of the three complexes on the α-glycosidase activity was determined using the *p*-nitrophenyl-D-glycopyranoside substrate, according to a previously reported procedure [77]. The three complexes showed inhibitory effects similar to the standard α-glycosidase inhibitor acarbose (IC_50_ = 49.61 µM and *K*_i_ = 55.84 ± 7.35 µM), being complex **24** the most active.

Deswal et al., (2022) [78] recently reported a study on transition metal complexes, namely cobalt (II), nickel(II), copper(II), and zinc(II), with SB ligands and their potential antidiabetic activity by in vitro experiments on α-amylase and α-glucosidase enzymes. Complexes **25** and **26** showed good activities with IC_50_ values close to those of the standard acarbose (IC_50_ = 1.28 ± 0.05 and 0.58 ± 0.06 μmol/mL, respectively). This outcome was supported by molecular docking studies carried out using the active site of the human pancreatic α-amylase (PDB code: 1BSI) and α-glucosidase (PDB code: 5ZCC). Moreover, in silico studies to check the drug-likeness were carried out, showing that the complexes can be used as orally active drugs. Table 2 summarizes the reported recent studies.

## 3. Binuclear SBs Complexes 

Binuclear complexes may present in the same or two different metals and are often more interesting than the mononuclear variants in chemical and biological studies. Binuclear complexes with SBs have been widely studied as catalysts [79,80,81], in fluorescence and luminescence studies [82,83], and also antimicrobial activities were reported [84,85]. More recently, binuclear complexes with SBs have been suggested for the therapy of COVID-19 disease [86] on the basis of molecular modeling studies [87].

Dicopper(II) complexes are often used to study catecholase (or catechol oxidase) activity, a type-3 copper protein with a dicopper(II) moiety. Catecholase can reversibly bind oxygen at room temperature, thus it can be used to oxidize phenols to the respective *o*-benzoquinones. Specifically, dicopper(II) complexes are often used to explore the catalytic activity of catecholase oxidation [88]. Recent studies on binuclear complexes with SBs are summarized in Table 3. 

Keypour et al., (2021) [89] reported the study of binuclear and mononuclear complexes and their cytotoxicity against breast (MCF-7) and lung (A549) adenocarcinoma cancer cell lines by the MTT assay. In general, they suggested that binuclear complexes possess a higher cytotoxic effect than the other mononuclear complexes and, specifically, the CuL1 (**27**) and NiL1 (**28**) complexes were the most active. 

Lei et al., (2022) [90] reported the study of a binuclear bismuth(III) complex [Bi_2_(HL)_2_(NO_3_)_4_] (**29**) with the SB ligand H_2_L, (*E*)-*N*’-(2-hydroxy-3-methoxybenzylidene)isonicotinohydrazide], and its antimicrobial activity against *Schizosaccharomyces pombe* ACCC 20047, determined by bio-microcalorimetry at 32 °C. X-ray crystallographic analysis revealed that the complex possessed a binuclear structure, with each bismuth(III) ion that is nine-coordinated by two tetradentate O_3_N-donor ligands and two nitrate ions. The antimicrobial activity of the complex is more pronounced than isoniazid, as obtained by comparing the IC_50_ values of the complex and the standard (IC_50_ = 1.63 × 10^−2^ mol/L).

Li et al., (2022) [91] described a study on the binuclear complex of bismuth(III) with (*E*)-*N’*-(2-hydroxy-4-methoxybenzylidene)isonicotinohydrazide (**30**) and the antimicrobial activity against two Gram-positive (*S. aureus* Newman and *B. subtilis* CMCC63501) and two Gram-negative (*E. coli* AB1157 and *P. aeruginosa* PA01) bacteria obtained from the China General Microbiological Culture Collection Center (CGMCC, Beijing, China) in comparison to three standards, namely, vancomycin, kanamycin, and tetracycline. The complex showed high activity against *S. aureus* vancomycin (MIC = 2 μg/mL and 1.54 μM), similar to that of vancomycin (MIC = 2 μg/mL and 1.38 μM). Moreover, antiproliferative studies were carried out against human gastric SNU-16 cancer cell lines procured from the ATCC by using Cell Counting Kit-8 (CCK-8). The complex showed activity similar to that of the standard doxorubicin (IC_50_ = 0.35 and 1.18 μM).

Kargar et al., (2022) [85] studied two binuclear zinc(II) SB complexes, Z1 (**31**) and Z2 (**32**). The antibacterial activity was evaluated against Gram-positive (*S. aureus* ATCC 25923 and *B. cereus* ATCC 11778), and Gram-negative (*E. coli* ATCC 25922 and *P. aeruginosa* ATCC 15442) bacteria by using the agar well diffusion method. The compounds showed activity against Gram-positive bacteria albeit lower than the standard, chloramphenicol (IZD = 30 mm for both *S. aureus* and *B. cereus*).

Goudarzi et al., (2022) [92] described two bi- and trinuclear palladium(II)-sodium complexes, {[PdL]Na(NO_3_)(EtOH)} (**33**) and {[PdL]_2_Na}Cl (**34**) based on salophen-type SB, *N*,*N*’-(1,2-phenylene)-bis(3-methoxysalicylideneimine). The antibacterial activity of the two complexes was studied against one Gram-negative strain (*E. coli* ATCC 25922) and one Gram-positive strain (*S. aureus* ATCC 6538) by the direct colony method. The two compounds were active as antibacterials, compared with the negative control (CFU/mL = 1.5 × 10^6^ against both bacteria strains). Complex **34** showed a higher anticancer activity against MCF-7 cell line, determined by the MTT assay, as well as a better antibacterial effect on the examined bacteria strains than complex **33**. Complexes **33** and **34** displayed high cytotoxicity, whereas carboplatin, used as a standard, exhibited no anticancer effect towards this cell line at the same concentration range within 24 h of incubation. 

Keshavarzian et al., (2022) [93] described a study on a dimetallic complex with copper (II) and gadolinium (III). The Cu(II)-Gd(III) SB complex (**35**) was studied for its binding affinity with FS-DNA, by several analytical methods, and intercalation was suggested as the main mode of binding. The interaction with Bovine serum albumin (BSA) was also studied demonstrating a strong complexation. Antiproliferative activity was carried out by the MTT assay towards MCF-7, HeLa, and human prostate cancer (LNCaP) cancer cell lines. Finally, the mechanism and kinetics of the catalytic activity of the complex in the oxidation reaction of 3,5-di-*tert*-butylcatechol, was studied at 298 K. The complex exhibited moderate catecholase activity in DMF medium.

Savcı et al., (2022) [94] described the study of three dinuclear metal complexes with SBs, with Cu(II), Ru(II), and Pd(II) as metals, and their antiproliferative activity against human colon cancer (Caco-2) and fibroblast cells (L-929) evaluated via the MTT assay, compared to the reference drug 5-fluorouracil (IC_50_ = 60.2 and 94.35 µM, respectively). The Cu(II) and Pd(II) complexes (**36** and **37**, respectively) showed a better cytotoxic effect against Caco-2 cells than the reference drug. Moreover, antioxidant studies were carried out via DPPH and ABTS assays, and the Pd(II) complex scavenged DPPH radicals close to the standard antioxidants (BHA, 70.19% and BHT, 44.71%), followed by the Cu(II) complex that scavenged the ABTS radicals in a better way (BHT, 85.16%; BHA, 84.7%). It is noteworthy that the antiproliferative effects of the complexes, as well as for the 5-fluorouracil, were significantly enhanced when used in combination with electroporation, resulting in a significant reduction of Caco-2 cancer cell viability.

Jahromi et al., (2022) [95] described a binuclear water-soluble Cu(II) complex [Cu^II^_2_L(*μ*_1,1_-NO_3_)(*μ*-OH)(NO_3_)(H_2_O), (**38**)], where L = [2,6-bis{*N*-(2-pyridylethyl)formidoyl}-4-methylphenol], which involves the μ-phenoxo N_2_O_4_ SB ligand. The structure was determined, and it was evidenced by binuclear Cu(II) ions located in a six-coordinated distorted environment. The interaction of the complex with FS-DNA/BSA was investigated by several analytical techniques, revealing that the complex interacted with DNA in both UV–Vis absorption and a competitive binding experiment with ethidium bromide and revealed an intercalation mode (*K*_b_ = 2.78 × 10^4^ M^−1^). In Tris-buffer, the complex emitted relatively weak luminescence and increasing DNA concentration, a remarkable increase in fluorescence intensity of about two folds and a strong green fluorescence was observed by the naked eye, demonstrating that the complex behaves like a “light switch” for DNA in water. The antiproliferative effect of the complex was studied against three cell lines, including LNCaP, HeLa, and MCF-7, by MTT assay and was time-dependent and significantly lowered after the treatment with the complex (in Table data after 72 h). Moreover, the efficiency of the complex was studied for catecholase activity toward the oxidation of 3,5-di-*tert*-butylcatechol (3,5-DTBC) to 3,5-di-*tert*-butylquinone (3,5-DTBQ) in acetonitrile and DMF by the UV–vis method. The complex showed high activity in catalyzing the oxidation of 3,5-DTBC to 3,5-DTBQ in acetonitrile.

## 4. Summary

SBs-metal complexes represent a fascinating and relevant research topic that continuously brings new information on newly synthesized compounds, which are usually inexpensive, easy to synthesize, and show numerous activities in a wide number of research fields. For instance, interesting biological activities have been found as antimicrobials, antiproliferative, and antioxidants, but other activities, such as anti-inflammatory, antimalarial, antidiabetic, and anti-Alzheimer have also been reported. The multiple activities are mostly due to the presence of the C=N linkage in the azomethine derivatives, but the wide range of applications is a consequence of the versatility of SBs reactions with several metals. This review presents an update of the most recent studies on mononuclear and binuclear metal complexes with SBs, focusing primarily on antimicrobial activity, which is one of the most important issues to date for overcoming antimicrobial resistance. Moreover, recent studies of these complexes as antimalarials and antidiabetics and as promising agents for the ever-present Alzheimer disease have also been described.

## Figures and Tables

**Table 2:** SBs mononuclear metal complexes with antimalarial, antioxidant, antidiabetic, and anti-Alzheimer activities.

Structure	Compound	% Inhibition, MIC, and IC_50_	*K*_i_	Ref
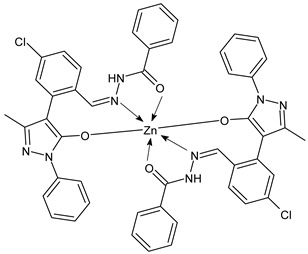	Zn(BZ-PCBPMP)2 (**20**)	MIC = 0.56 μmol/L (*P. falciparum*)	-	Shaikh et al., 2022 [74]
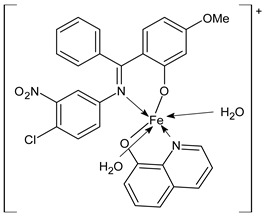	Fe(III) complex (**21**)	% inhib: 65.54% (antimalarial) % inhib: 52–54% (α-amylase) % inhib: 94.27% (DPPH)	-	Ragole et al., 2022 [75]
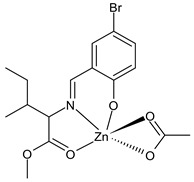	**22**	IC_50_ = 12.45 µM (AChE) IC_50_ = 9.61 µM (BChE) IC_50_ = 35.57 µM (α-glycosidase)	*K*_i_ = 11.92 ± 3.90 µM (AChE) *K*_i_ = 6.98 ± 0.96 µM (BChE) *K*_i_ = 38.17 ± 6.26 µM (α-glycosidase)	Şenocak et al., 2021 [76]
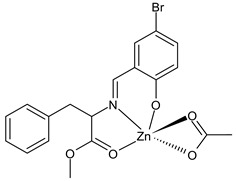	**23**	IC_50_ = 7.24 µM (AChE) IC_50_ = 4.73 µM (BChE) IC_50_ = 30.84 µM (α-glycosidase)	*K*_i_ = 6.26 ± 0.83 µM (AChE) *K*_i_ = 3.68 ± 0.54 µM (BChE) *K*_i_ = 37.14 ± 7.12 µM (α-glycosidase)	Şenocak et al., 2021 [76]
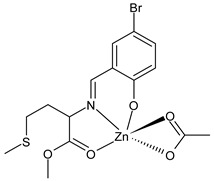	**24**	IC_50_ = 18.06 µM (AChE) IC_50_ = 11.25 µM (BChE) IC_50_ = 26.21 µM (α-glycosidase)	*K*_i_ = 15.73 ± 4.73 µM (AChE) *K*_i_ = 10.27 ± 1.68 µM (BChE) *K*_i_ = 30.50 ± 3.82 µM (α-glycosidase)	Şenocak et al., 2021 [76]
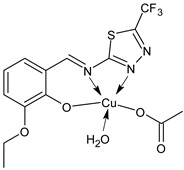	**25**	IC_50_ = 1.41 ± 0.03 μmol/mL (α-amylase) IC_50_ = 0.62 ± 0.07 μmol/mL (α-glucosidase)	-	Deswal et al., 2022 [78]
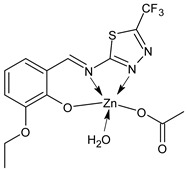	**26**	IC_50_ = 1.33 ± 0.05 μmol/mL (α-amylase) IC_50_ = 0.60 ± 0.05 μmol/mL (α-glucosidase)	-	Deswal et al., 2022 [78]

**Table 3:** Binuclear metal complexes with SBs.

Structure	Compound	Antimicrobial and Antioxidant Activities	IC_50_	Ref
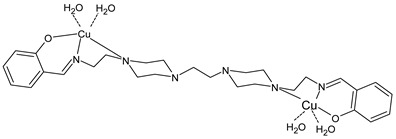	CuL1 (**27**)	-	IC_50_ = 9.48 ± 0.24 µM (MCF-7) IC_50_ = 10.15 ± 0.6 µM (A549)	Keypour et al., 2021 [89]
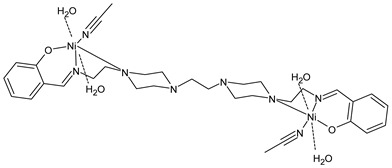	NiL1 (**28**)	-	IC_50_ = 12.16 ± 0.79 µM (MCF-7) IC_50_ = 13.63 ± 0.91 µM (A549)	Keypour et al., 2021 [89]
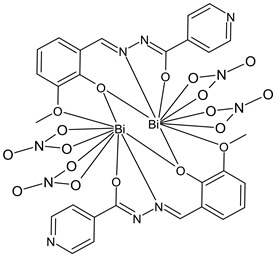	Bi_2_(HL)_2_(NO_3_)_4_ (**29**)	-	IC_50_ = 3.93 × 10^−4^ mol/L (*S. pombe*)	Lei et al., 2022 [90]
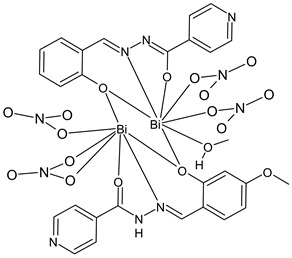	(**30**)	MIC = 2 μg/mL and 1.38 μM (*S. aureus* Newman)	IC_50_ = 0.23 ± 0.09 μM (SNU-16)	Li et al., 2022 [91]
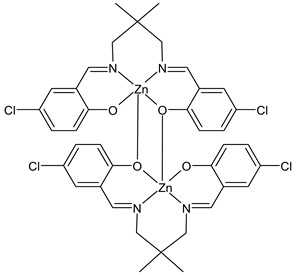	Z1 (**31**)	IZD = 16 mm (*S. aureus* PTCC1431) IZD = 15 mm (*B. cereus* PTCC1015)	-	Kargar et al., 2022 [85]
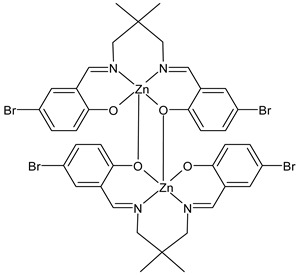	Z2 (**32**)	IZD = 11 mm (*S. aureus* PTCC1431) IZD = 11 mm (*B. cereus* PTCC1015)	-	Kargar et al., 2022 [85]
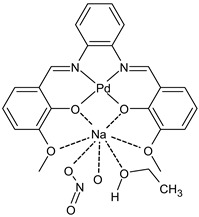	**33**	CFU/mL = 2.2 × 10^5^ (*E. coli* ATCC 25922); Antibacterial rate = 85% CFU/mL = 6.0 × 10^5^ (*S. aureus* ATCC 6538); Antibacterial rate = 88%	IC_50_ = 103 μM (MCF-7)	Goudarzi et al., 2022 [92]
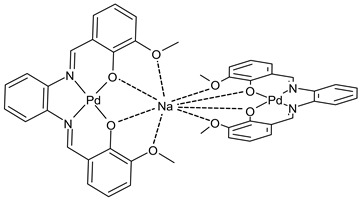	**34**	CFU/mL = 1.8 × 10^5^ (*E. coli* ATCC 25922); Antibacterial rate = 85% CFU/mL = 5.1 × 10^5^ (*S. aureus* ATCC 6538); Antibacterial rate = 66%	IC_50_ = 89 μM (MCF-7)	Goudarzi et al., 2022 [92]
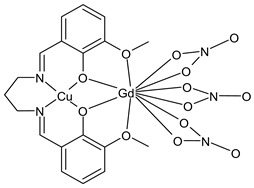	Cu-Gd complex (**35**)	-	IC_50_ = 36.09 ± 4.11 µM (MCF-7) IC_50_ = 15.66 ± 2.92 (HeLa) IC_50_ = 20.23 ± 4.77 (LNCaP) IC_50_ = 96.62 ± 3.11 (normal fibroblasts)	Keshavarzian et al., 2022 [93]
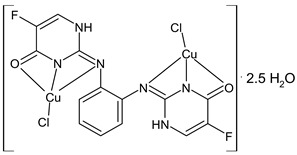	Cu(II) complex (**36**)	Scavenging inhibition (%) = 40.92% (DPPH) scavenging inhibition (%) = 87.12% (ABTS)	IC_50_ = 31.88 μM (Caco-2) IC_50_ = 285 μM (L-929)	Savcı et al., 2022 [94]
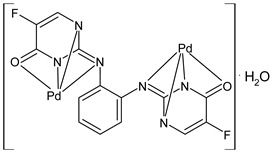	Pd(II) complex (**37**)	Scavenging inhibition (%) = 80.48% (DPPH) scavenging inhibition (%) = 43.15% (ABTS)	IC_50_ = 25.35 μM (Caco-2) IC_50_ = 114.28 μM (L-929)	Savcı et al., 2022 [94]
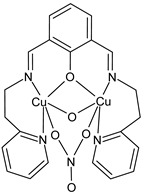	Cu^II^_2_L(*μ*_1,1_-NO_3_)(*μ*-OH)(NO_3_)(H_2_O) (**38**)	-	IC_50_ = 49.92 ± 4.90 µmol/L (MCF-7) IC_50_ = 21.53 ± 2.72 µmol/L (HeLa) IC_50_ = 29.32 ± 5.42 µmol/L (LNCaP) IC_50_ = 73.03 ± 4.16 µmol/L (Normal Skin Fibroblasts)	Jahromi et al., 2022 [95]

## Data Availability

Not applicable.

## References

[1] Mondal K., Mistri S. (2022). Schiff base based metal complexes: A review of their catalytic activity on aldol and henry reaction. Comm. Inorg. Chem..

[2] Mukhtar S.S., Hassan A.S., Morsy N.M., Hafez T.S., Hassaneen H.M., Saleh F.M. (2021). Overview on synthesis, reactions, applications, and biological activities of Schiff bases. Egypt. J. Chem..

[3] Nath B.D., Islam M., Karim R., Rahman S., Shaikh A.A., Georghiou P.E., Menelaou M. (2022). Recent progress in metal-incorporated acyclic Schiff-base derivatives: Biological aspects. Chem. Sel..

[4] Ceramella J., Iacopetta D., Catalano A., Cirillo F., Lappano R., Sinicropi M.S. (2022). A review on the antimicrobial activity of Schiff bases: Data collection and recent studies. Antibiotics.

[5] Iacopetta D., Ceramella J., Catalano A., Saturnino C., Bonomo M.G., Franchini C., Sinicropi M.S. (2021). Schiff bases: Interesting scaffolds with promising antitumoral properties. Appl. Sci..

[6] Ibezim A., Ofokansi M.N., Ndukwe X., Chiama C.S., Obi B.C., Isiogugu O.N., Ikechukwu P.E., Onwuka A.M., Ihim S.A., Asegbeloyin J.N. (2022). Evaluation of anti-malarial potency of new pyrazole-hydrazine coupled to Schiff base derivatives. Malaria J..

[7] Tople M.S., Patel N.B., Patel P.P., Purohit A.C., Ahmad I., Patel H. (2023). An in silico-in vitro antimalarial and antimicrobial investigation of newer 7-chloroquinoline based Schiff-bases. J. Mol. Struct..

[8] Tokali F.S., Taslimi P., Usanmaz H., Karaman M., Sendil K. (2021). Synthesis, characterization, biological activity and molecular docking studies of novel Schiff bases derived from thiosemicarbazide: Biochemical and computational approach. J. Mol. Struct..

[9] Goleij M., Youseftabar-Miri L., Montazeri M., Khakpai F. (2021). Induction of anxiolytic, antidepressant and analgesic effects by Shiff base of (*E*)-3-(1*H*-imidazol-4-yl)-2-((2-oxoindolin-3-ylidene) amino) propanoic acid derivatives in diabetic rats. J. Diab. Metab. Disord..

[10] Arora R., Sharma R., Tageza A., Grewal A.S., Saini B., Arora S., Kaur R. (2021). Design and synthesis of novel 4-aminophenazone Schiff bases by grinding technique as prospective anti-inflammatory agents. J. Appl. Pharm. Sci..

[11] Hamid S.J., Salih T. (2022). Design, synthesis, and anti-inflammatory activity of some coumarin Schiff base derivatives: In silico and in vitro study. Drug Des. Develop. Ther..

[12] Alotaibi S.H., Amer H.H., Touil N., Abdel-Moneim A.S., Soliman M.M., Zaki Y.H. (2022). Synthesis, characterization and molecular docking of new nucleosides and Schiff bases derived from ampyrone as antiviral agents to contain the COVID-19 virus. Polycycl. Arom. Comp..

[13] Varpe B.D., Jadhav S.B. (2022). Schiff base of isatin with 2-thiopheneethylamine and its Mannich bases: Synthesis, docking, and in vitro anti-inflammatory and antitubercular activity. Russ. J. Bioorg. Chem..

[14] Hamad A., Chen Y., Khan M.A., Jamshidi S., Saeed N., Clifford M., Hind C., Sutton J.M., Rahman K.M. (2021). Schiff bases of sulphonamides as a new class of antifungal agent against multidrug-resistant *Candida auris*. Microbiol. Open.

[15] Taha M., Rahim F., Zaman K., Anouar E.H., Uddin N., Nawaz F., Sajid M., Khan K.M., Shah A.A., Wadood A. (2021). Synthesis, in vitro biological screening and docking study of benzo[*d*]oxazole bis Schiff base derivatives as a potent anti-Alzheimer agent. J. Biomol. Struct. Dynam..

[16] Boulguemh I.E., Beghidja A., Khattabi L., Long J., Beghidja C. (2020). Monomeric and dimeric copper(II) complexes based on bidentate *Nʹ*-(propan-2-ylidene)thiophene carbohydrazide Schiff base ligand: Synthesis, structure, magnetic properties, antioxidant and anti-Alzheimer activities. Inorg. Chim. Acta.

[17] Hameed A., Al-Rashida M., Uroos M., Ali S.A., Khan K.M. (2017). Schiff bases in medicinal chemistry: A patent review (2010–2015). Expert Opin. Ther. Pat..

[18] Afzal H.R., Khan N.U.H., Sultana K., Mobashar A., Lareb A., Khan A., Gull A., Afzaal H., Khan M.T., Rizwan M. (2021). Schiff bases of pioglitazone provide better antidiabetic and potent antioxidant effect in a streptozotocin-nicotinamide-induced diabetic rodent model. ACS Omega.

[19] Daoud S., Thiab S., Jazzazi T.M.A., Alshboul T.M.A., Ullah S. (2022). Evaluation and molecular modelling of bis-Schiff base derivatives as potential leads for management of diabetes mellitus. Acta Pharm..

[20] Jamil W., Shaikh J., Yousuf M., Taha M., Khan K.M., Shah S.A.A. (2021). Synthesis, anti-diabetic and in silico QSAR analysis of flavone hydrazide Schiff base derivatives. J. Biomol. Struct. Dyn..

[21] Wang J., Li N., Yang X.Q., Wang L.L., Ni R.Y., He Y.N., Zhang C. (2022). A highly selective turn-on Schiff base fluorescent sensor for diabetic biomarker beta-hydroxybutyrate (β-HB). Dyes Pigments.

[22] Soroceanu A., Bargan A. (2022). Advanced and biomedical applications of Schiff-base ligands and their metal complexes: A review. Crystals.

[23] Hossain A.M.S., Méndez-Arriaga J.M., Xia C., Xie J., Gómez-Ruiz S. (2022). Metal complexes with ONS donor Schiff bases. Polyhedron.

[24] Kaur M., Kumar S., Younis S.A., Yusuf M., Lee J., Weon S., Kim K.-H., Malik A.K. (2021). Post-Synthesis modification of metal-organic frameworks using Schiff base complexes for various catalytic applications. Chem. Eng. J..

[25] Kargar H., Nateghi-Jahromi M., Fallah-Mehrjardi M., Behjatmanesh-Ardakani R., Munawar K.S., Ali S., Ashfaq M., Tahir M.N. (2022). Synthesis, spectral characterization, crystal structure and catalytic activity of a novel dioxomolybdenum Schiff base complex containing 4-aminobenzhydrazone ligand: A combined experimental and theoretical study. J. Mol. Struct..

[26] Khashei Siuki H., Ghamari Kargar P., Bagherzade G. (2022). New acetamidine Cu(II) Schiff base complex supported on magnetic nanoparticles pectin for the synthesis of triazoles using click chemistry. Sci. Rep..

[27] Kargar H., Meybodi F.A., Ardakani R.B., Elahifard M.R., Torabi V., Mehrjardi M.F., Tahir M.N., Ashfaq M., Munaware K.S. (2021). Synthesis, crystal structure, theoretical calculation, spectroscopic and antibacterial activity studies of copper (II) complexes bearing bidentate Schiff base ligands derived from 4-aminoantipyrine: Influence of substitutions on antibacterial activity. J. Mol. Struct..

[28] Kargar H., Aghaei-Meybodi F., Elahifard M.R., Tahir M.N., Ashfaq M., Munawar K.S. (2021). Some new Cu(II) complexes containing O, N-donor Schiff base ligands derived from 4-aminoantipyrine: Synthesis, characterization, crystal structure and substitution effect on antimicrobial activity. J. Coord. Chem..

[29] Ghobakhloo F., Azarifar D., Mohammadi M., Keypour H., Zeynali H. (2022). Copper(II) Schiff-base complex modified UiO-66-NH_2_ (Zr) metal–organic framework catalysts for Knoevenagel condensation–Michael addition–cyclization reactions. Inorg. Chem..

[30] Sahraei A., Kargar H., Hakimi M., Tahir M.N. (2017). Synthesis, characterization, crystal structures and biological activities of eight-coordinate zirconium(IV) Schiff base complexes. Transit. Metal Chem..

[31] Sahraei A., Kargar H., Hakimi M., Tahir M.N. (2017). Distorted square-antiprism geometry of new zirconium(IV) Schiff base complexes: Synthesis, spectral characterization, crystal structure and investigation of biological properties. J. Mol. Struct..

[32] Kargar H., Fallah-Mehrjardi M., Behjatmanesh-Ardakani R., Bahadori M., Moghadam M., Ashfaq M., Munawar K.S., Tahir M.N. (2022). Spectroscopic investigation, molecular structure, catalytic activity with computational studies of a novel Pd (II) complex incorporating unsymmetrical tetradentate Schiff base ligand. Inorg. Chem. Comm..

[33] Rahmatpour F., Kosari M., Monadi N. (2022). Catalytic performance of copper(II) Schiff base complex immobilized on Fe_3_O_4_ nanoparticles in synthesis of 2-amino-4*H*-benzo[*h*]chromenes and reduction of 4-nitrophenol. J. Mol. Struct..

[34] Ourari A., Aggoun D., Karce H.E., Berenguer R., Morallon E., Lanez T., Ouennoughi Y. (2022). Electrochemistry and study of indirect electrocatalytic properties of a novel organometallic Schiff base nickel(II) complex. J. Organometal. Chem..

[35] Chen D., Han C. (2022). An alkoxo-bridged dinuclear ruthenium-Schiff base complex: Synthesis, structure and catalytic reactivity. Inorg. Chem. Comm..

[36] Jasim S.A., Riadi Y., Majdi H.S., Altimari U.S. (2022). Nanomagnetic macrocyclic Schiff-base–Mn(II) complex: An efficient heterogeneous catalyst for click approach synthesis of novel β-substitued-1,2,3-triazoles. RSC Adv..

[37] Kargar H., Fallah-Mehrjardi M., Ashfaq M., Munawar K.S., Tahir M.N., Behjatmanesh-Ardakani R., Rudbari H.A., Ardakani A.A., Sedighi-Khavidak S. (2021). Zn(II) complexes containing O,N,N,O-donor Schiff base ligands: Synthesis, crystal structures, spectral investigations, biological activities, theoretical calculations and substitution effect on structures. J. Coord. Chem..

[38] Kargar H., Fallah-Mehrjardi M., Behjatmanesh-Ardakani R., Rudbari H.A., Ardakani A.A., Sedighi-Khavidak S., Munawarf K.S., Ashfaq M., Tahir M.N. (2022). Synthesis, spectral characterization, crystal structures, biological activities, theoretical calculations and substitution effect of salicylidene ligand on the nature of mono and dinuclear Zn(II) Schiff base complexes. Polyhedron.

[39] Kargar H., Moghadam M., Shariati L., Feizi N. (2022). Novel oxo–peroxo W(VI) Schiff base complex: Synthesis, SC-XRD, spectral characterization, supporting on chloromethylated polystyrene, and catalytic oxidation of sulfides. J. Iran. Chem. Soc..

[40] Oliveri I.P., Consiglio G., Munzi G., Failla S. (2022). Deaggregation properties and transmetalation studies of a zinc(II) salen-type Schiff-base complex. Dalton Transact..

[41] Oliveri I.P., Munzi G. (2022). A simple approach based on transmetalation for the selective and sensitive colorimetric/fluorometric detection of copper(II) ions in drinking water. New J. Chem..

[42] Abd El-Lateef H.M., Mohamad A.D.M., Shehata M.R., Abu-Dief A.M. (2022). Targeted synthesis of two iron(III) tetradentate dibasic chelating Schiff base complexes toward inhibition of acidic induced steel corrosion: Empirical and DFT insights. Appl. Organometal. Chem..

[43] Liu X., Yang X., Ma Y., Liu J., Shi D., Niu M., Schipper D. (2021). Construction of two lanthanide Schiff base complexes: Chiral “triple-decker” structure and NIR luminescent response towards anions. J. Luminesc..

[44] Chi S., Xu Y., Xie B., Gao T. (2022). Luminescence of Zn-Yb dinuclear Schiff base complex: Enhanced NIR emission by modification with larger conjugated light-harvesting moieties. J. Mol. Struct..

[45] Kornikov A.I., Mustakimov R.E., Goloveshkin A.S., Tcelykh L.O., Vashchenko A.A., Medvedko A.V., Lepnev L.S., Utochnikova V.V. (2022). Novel ytterbium Schiff base complex: Toward efficient solution-processed NIR-emitting OLED. Org. Electron..

[46] Dong J., Wan T., Li K., Kong X., Shen Q., Wu H. (2022). Mononuclear Dy(III)/heteropolynuclear Zn(II)–Dy(III) Schiff base complexes: Synthesis, structures, fluorescence and antioxidant properties. J. Mol. Struct..

[47] Malik U.S., Niazi M.B.K., Jahan Z., Zafar M.I., Vo D.-V.N., Sher F. (2022). Nano-structured dynamic Schiff base cues as robust self-healing polymers for biomedical and tissue engineering applications: A review. Environ. Chem. Lett..

[48] Rezayati S., Ramazani A., Sajjadifar S., Aghahosseini H., Rezaei A. (2021). Design of a Schiff base complex of copper coated on epoxy-modified core–shell MNPs as an environmentally friendly and novel catalyst for the one-pot synthesis of various chromene-annulated heterocycles. ACS Omega.

[49] Biswas B., Choudhury P., Ghosh A., Dubey S.K., Rizzoli C., Saha R., Bhattacharjee S. (2022). A water soluble Ni-Schiff base complex for homogeneous green catalytic CS cross-coupling reactions. Inorg. Chim. Acta.

[50] Lashanizadegan M., Gorgannejad Z., Sarkheil M. (2021). Cu(II) Schiff base complex on magnetic support: An efficient nano-catalyst for oxidation of olefins using H_2_O_2_ as an eco-friendly oxidant. Inorg. Chem. Commun..

[51] Aragón-Muriel A., Reyes-Márquez V., Cañavera-Buelvas F., Parra-Unda J.R., Cuenú-Cabezas F., Polo-Cerón D., Colorado-Peralta R., Suárez-Moreno G.V., Aguilar-Castillo B.A., Morales-Morales D. (2022). Pincer complexes derived from tridentate Schiff bases for their use as antimicrobial metallopharmaceuticals. Inorganics.

[52] Aggarwal N., Maji S. (2022). Potential applicability of Schiff bases and their metal complexes during COVID-19 pandemic–A review. Rev. Inorg. Chem..

[53] Jain S., Rana M., Sultana R., Mehandi R., Rahisuddin (2022). Schiff base metal complexes as antimicrobial and anticancer agents. Polycycl. Arom. Comp..

[54] Ghanghas P., Choudhary A., Kumar D., Poonia K. (2021). Coordination metal complexes with Schiff bases: Useful pharmacophores with comprehensive biological applications. Inorg. Chem. Commun..

[55] Kargar H., Behjatmanesh-Ardakani R., Torabi V., Kashani M., Chavoshpour-Natanzi Z., Kazemi Z., Mirkhani V., Sahraei A., Tahir M.N., Ashfaq M. (2021). Synthesis, characterization, crystal structures, DFT, TD-DFT, molecular docking and DNA binding studies of novel copper(II) and zinc(II) complexes bearing halogenated bidentate N,O-donor Schiff base ligands. Polyhedron.

[56] Kargar H., Behjatmanesh-Ardakani R., Torabi V., Sarvian A., Kazemi Z., Chavoshpour-Natanzi Z., Mirkhani V., Sahraei A., Tahir M.N., Ashfaq M. (2021). Novel copper(II) and zinc(II) complexes of halogenated bidentate N, O-donor Schiff base ligands: Synthesis, characterization, crystal structures, DNA binding, molecular docking, DFT and TD-DFT computational studies. Inorg. Chim. Acta.

[57] Kar K., Ghosh D., Kabi B., Chandra A. (2022). A concise review on cobalt Schiff base complexes as anticancer agents. Polyhedron.

[58] Catalano A., Sinicropi M.S., Iacopetta D., Ceramella J., Mariconda A., Rosano C., Scali E., Saturnino C., Longo P. (2021). A review on the advancements in the field of metal complexes with Schiff bases as antiproliferative agents. Appl. Sci..

[59] Catalano A., Iacopetta D., Ceramella J., Scumaci D., Giuzio F., Saturnino C., Aquaro S., Rosano C., Sinicropi M.S. (2022). Multidrug resistance (MDR): A widespread phenomenon in pharmacological therapies. Molecules.

[60] Rajakkani P., Alagarraj A., Thangavelu S.A.G. (2021). Tetraaza macrocyclic Schiff base metal complexes bearing pendant groups: Synthesis, characterization and bioactivity studies. Inorg. Chem. Commun..

[61] Abu-Dief A.M., El-Khatib R.M., Aljohani F.S., Alzahrani S.O., Mahran A., Khalifa M.E., El-Metwaly N.M. (2021). Synthesis and intensive characterization for novel Zn(II), Pd(II), Cr(III) and VO(II)-Schiff base complexes; DNA-interaction, DFT, drug-likeness and molecular docking studies. J. Mol. Struct..

[62] Kargar H., Ardakani A.A., Tahir M.N., Ashfaq M., Munawar K.S. (2021). Synthesis, spectral characterization, crystal structure and antibacterial activity of nickel(II), copper(II) and zinc(II) complexes containing ONNO donor Schiff base ligands. J. Mol. Struct..

[63] Kargar H., Ashfaq M., Fallah-Mehrjardi M., Behjatmanesh-Ardakani R., Munawar K.S., Tahir M.N. (2022). Unsymmetrical Ni(II) Schiff base complex: Synthesis, spectral characterization, crystal structure analysis, Hirshfeld surface investigation, theoretical studies, and antibacterial activity. J. Mol. Struct..

[64] Al-Shboul T.M., El-khateeb M., Obeidat Z.H., Ababneh T.S., Al-Tarawneh S.S., Al Zoubi M.S., Alshaer W., Abu Seni A., Qasem T., Moriyama H. (2022). Synthesis, characterization, computational and biological activity of some Schiff bases and their Fe, Cu and Zn complexes. Inorganics.

[65] Sirignano E., Saturnino C., Botta A., Sinicropi M.S., Caruso A., Pisano A., Lappano R., Maggiolini M., Longo P. (2013). Synthesis, characterization and cytotoxic activity on breast cancer cells of new half-titanocene derivatives. Bioorg. Med. Chem. Lett..

[66] Maia D.O., Santos V.F., Barbosa C.R., Fróes Y.N., Muniz D.F., Santos A.L., Santos M.H.C., Silva R.R.S., Silva C.G.L., Souza R.O.S. (2022). Nickel(II) chloride Schiff base complex: Synthesis, characterization, toxicity, antibacterial and leishmanicidal activity. Chem. Biol. Interact..

[67] Tyula Y.A., Goudarziafshar H., Yousefi S., Dušek M., Eigner V. (2023). Template synthesis, characterization and antibacterial activity of d10 (Zn^2+^, Cd^2+^, Hg^2+^) Schiff base complexes: A novel supramolecular Cd^2+^ complex with two 1D helical chains, and its Hirshfeld surface analysis. J. Mol. Struct..

[68] Saritha T.J., Metilda P. (2021). Synthesis, spectroscopic characterization and biological applications of some novel Schiff base transition metal(II) complexes derived from curcumin moiety. J. Saudi Chem. Soc..

[69] Belaid S., Landreau A., Djebbar S., Benali-Baitich O., Bouet G., Bouchara J.P. (2008). Synthesis, characterization and antifungal activity of a series of manganese(II) and copper(II) complexes with ligands derived from reduced N, N′-O-phenylenebis (salicylideneimine). J. Inorg. Biochem..

[70] Dharmaraj N., Viswanathamurthi P., Natarajan K. (2001). Ruthenium(II) complexes containing bidentate Schiff bases and their antifungal activity. Transit. Metal Chem..

[71] Mizushima Y., Kobayashi M. (1968). Interaction of anti-inflammatory drugs with serum proteins, especially with some biologically active proteins. J. Pharm. Pharmacol..

[72] Gowdhami B., Manojkumar Y., Vimala R.T.V., Ramya V., Karthiyayini B., Kadalmani B., Akbarsha M.A. (2022). Cytotoxic cobalt(III) Schiff base complexes: In vitro anti-proliferative, oxidative stress and gene expression studies in human breast and lung cancer cells. BioMetals.

[73] Hassona S.M., Saad E.A., Kiwan H.A., Hassanien M.M. (2022). Palladium(II) Schiff base complex arrests cell cycle at early stages, induces apoptosis, and reduces Ehrlich solid tumor burden: A new candidate for tumor therapy. Invest. New Drugs.

[74] Shaikh I., Travadi M., Jadeja R.N., Butcher R.J., Pandya J.H. (2022). Crystal feature and spectral characterization of Zn (II) complexes containing Schiff base of Acylpyrazolone ligand with antimalarial action. J. Indian Chem. Soc..

[75] Ragole V.D., Gayakwad S.V., Wankhede D.S. (2022). Novel Schiff base (*E*)-2-((4-chloro-3-nitrophenylimino)(phenyl)methyl)-5-methoxyphenol and mixed ligand complexes of Mn (II), Fe(III), Co(II), Ni(II) and Cu(II): Synthesis, structure elucidation and potency study as antibacterial, antimalarial, antioxidant, antidibetic and anticancer agents. J. Iran. Chem. Soc..

[76] Şenocak A., Taş N.A., Taslimi P., Tüzün B., Aydin A., Karadağ A. (2022). Novel amino acid Schiff base Zn(II) complexes as new therapeutic approaches in diabetes and Alzheimer’s disease: Synthesis, characterization, biological evaluation, and molecular docking studies. J. Biochem. Mol. Toxicol..

[77] Tao Y., Zhang Y.F., Cheng Y.Y., Wang Y. (2013). Rapid screening and identification of α-glucosidase inhibitors from mulberry leaves using enzyme-immobilized magnetic beads coupled with HPLC/MS and NMR. Biomed. Chromatogr..

[78] Deswal Y., Asija S., Dubey A., Deswal L., Kumar D., Jindal D.K., Devi J. (2022). Cobalt(II), nickel(II), copper(II) and zinc(II) complexes of thiadiazole based Schiff base ligands: Synthesis, structural characterization, DFT, antidiabetic and molecular docking studies. J. Mol. Struct..

[79] Sardarian A.R., Kazemnejadi M., Esmaeilpour M. (2021). Functionalization of superparamagnetic Fe_3_O_4_@SiO_2_ nanoparticles with a Cu (II) binuclear Schiff base complex as an efficient and reusable nanomagnetic catalyst for *N*-arylation of α-amino acids and nitrogen-containing heterocycles with aryl halides. Appl. Organometal. Chem..

[80] El-Gammal O.A., Saad D.A., Al-Hossainy A.F. (2021). Synthesis, spectral characterization, optical properties and X-ray structural studies of S centrosymmetric N_2_S_2_ or N_2_S_2_O_2_ donor Schiff base ligand and its binuclear transition metal complexes. J. Mol. Struct..

[81] Banerjee A., Das D., Ray P.P., Banerjee S., Chattopadhyay S. (2021). Phenoxo-bridged dinuclear mixed valence cobalt(III/II) complexes with reduced Schiff base ligands: Synthesis, characterization, band gap measurements and fabrication of Schottky barrier diodes. Dalton Transact..

[82] Yin J., Zhang X.M., Zhang X.M., Gao H.L., Cui J.Z. (2021). Near-infrared luminescence and magnetism of dinuclear lanthanide complexes constructed from a Schiff-base and different β-diketonate coligands. Inorg. Chim. Acta.

[83] Barwiolek M., Jankowska D., Chorobinski M., Kaczmarek-Kędziera A., Łakomska I., Wojtulewski S., Muzioł T.M. (2021). New dinuclear zinc(II) complexes with Schiff bases obtained from o-phenylenediamine and their application as fluorescent materials in spin coating deposition. RSC Adv..

[84] Goudarziafshar H., Yousefi S., Tyula Y.A., Dušek M., Eigner V. (2022). Template synthesis, DNA binding, antimicrobial activity, Hirshfeld surface analysis, and 1D helical supramolecular structure of a novel binuclear copper(II) Schiff base complex. RSC Adv..

[85] Kargar H., Fallah-Mehrjardi M., Behjatmanesh-Ardakani R., Rudbari H.A., Ardakani A.A., Sedighi-Khavidak S., Munawar K.S., Ashfaq M., Tahir M.N. (2022). Binuclear Zn(II) Schiff base complexes: Synthesis, spectral characterization, theoretical studies and antimicrobial investigations. Inorg. Chim. Acta.

[86] Iacopetta D., Ceramella J., Catalano A., Saturnino C., Pellegrino M., Mariconda A., Longo P., Sinicropi M.S., Aquaro S. (2022). COVID-19 at a glance: An up-to-date overview on variants, drug design and therapies. Viruses.

[87] Refat M.S., Gaber A., Alsanie W.F., Kobeasy M.I., Zakaria R., Alam K. (2021). Utilization and simulation of innovative new binuclear Co(II), Ni(II), Cu(II), and Zn(II) diimine Schiff base complexes in sterilization and coronavirus resistance (COVID-19). Open Chem..

[88] Salunke P.S., Puranik A.A., Kulkarni N.D. (2022). Histamine derived dimer of µ-chlorido-µ-phenoxido dicopper(II) complex as a potential enzyme mimic with catecholase activity. Polyhedron.

[89] Keypour H., Forouzandeh F., Hajari S., Jamshidi M., Farida S.H.M., Gable R.W. (2021). Synthesis, characterization, in vitro cytotoxicity activity, and molecular docking studies of mononuclear and binuclear macroacyclic Schiff base complexes. Polyhedron.

[90] Lei Y.H., Jiang J.H., Li X., Li Q.G., Li C.H. (2022). A nine-coordinated bismuth(III) Schiff-base complex: Design, synthesis, computational studies, and antimicrobial activity. Appl. Organometal. Chem..

[91] Li C.H., Ji M.H., Zhang K.W., Sun S.Y., Jiang J.H. (2022). Dinuclear bismuth (III) complex constructed by isoniazid-derived Schiff-base: Synthesis, crystal structure, and biological activity. Appl. Organometal. Chem..

[92] Goudarzi A., Saeidifar M., Aghapoor K., Mohsenzadeh F., Fenske D., Fuhr O., Ghassemzadeh M. (2022). Unprecedented bi-and trinuclear palladium(II)-sodium complexes from a salophen-type Schiff base: Synthesis, characterization, thermal behavior, and in vitro biological activities. J. Mol. Struct..

[93] Keshavarzian E., Asadi Z., Poupon M., Dusek M., Rastegari B. (2022). Heterodinuclear Cu–Gd (3d-4f) complex with di-compartmental Schiff base ligand in biological activity: Synthesis, crystal structure, catecholase activity and DNA & BSA-binding studies. J. Mol. Liq..

[94] Savcı A., Turan N., Buldurun K., Alkış M.E., Alan Y. (2022). Schiff base containing fluorouracil and its M(II) complexes: Synthesis, characterization, cytotoxic and antioxidant activities. Inorg. Chem. Commun..

[95] Jahromi Z.M., Asadi Z., Eigner V., Dusek M., Rastegari B. (2022). A new phenoxo-bridged dicopper Schiff base complex: Synthesis, crystal structure, DNA/BSA interaction, cytotoxicity assay and catecholase activity. Polyhedron.

